# A retrospective analysis of 12,400 SARS-CoV-2 RNA tests in patients with COVID-19 in Wuhan

**DOI:** 10.1097/MD.0000000000025916

**Published:** 2021-05-21

**Authors:** Jingwei Wang, Anyu Bao, Jian Gu, Xiaoyun He, Zegang Wu, Bin Qiao, Zhen Chen, Liang Xiong, Yan Zhang, Hongyun Zheng, Feng Li, Zhijun Zhao, Siqing Mei, Yongqing Tong

**Affiliations:** a Department of Clinical Laboratory, Renmin Hospital of Wuhan University, Wuhan; b Clinical Laboratory Center & Ningxia Key Laboratory of Clinical and Pathogenic Microbiology, General Hospital of Ningxia Medical University, Yinchuan, China.

**Keywords:** COVID-19, RT-qPCR, SARS-CoV-2, type of specimen

## Abstract

The outbreak and widely spread of coronavirus disease 2019 (COVID-19) has become a global public health concern. COVID-19 has caused an unprecedented and profound impact on the whole world, and the prevention and control of COVID-19 is a global public health challenge remains to be solved. The retrospective analysis of the large scale tests of SARS-CoV-2 RNA may indicate some important information of this pandemic. We selected 12400 SARS-CoV-2 tests detected in Wuhan in the first semester of 2020 and made a systematic analysis of them, in order to find some beneficial clue for the consistent prevention and control of COVID-19.

SARS-CoV-2 RNA was detected in suspected COVID-19 patients with real-time fluorescence quantitative PCR (RT-qPCR). The patients’ features including gender, age, type of specimen, source of patients, and the dynamic changes of the clinical symptoms were recorded and statistically analyzed. Quantitative and qualitive statistical analysis were carried out after laboratory detection.

The positive rate of SARS-CoV-2 was 33.02% in 12,400 suspected patients’ specimens in Wuhan at the first months of COVID-19 epidemics. SARS-CoV-2 RT-qPCR test of nasopharyngeal swabs might produce 4.79% (594/12400) presumptive results. The positive rate of SARS-CoV-2 RNA was significantly different between gender, age, type of specimen, source of patients, respectively (*P* < .05). The median window period from the occurrence of clinical symptom or close contact with COVID-19 patient to the first detection of positive PCR was 2 days (interquartile range, 1–4 days). The median interval time from the first SARS-CoV-2 positive to the turning negative was 14 days (interquartile range, 8–19.25 days).

This study reveals the comprehensive characteristics of the SARS-CoV-2 RNA detection from multiple perspectives, and it provides important clues and may also supply useful suggestions for future work of the prevention and treatment of COVID-19.

## 1 Introduction

Coronavirus disease 2019 (COVID-19) was reported in Wuhan, Hubei Province of China in early 2020.^[1]^ The pathogen of COVID-19 has been isolated and identified as a novel coronavirus on January 7, 2020,^[2]^ and was later named by the International Virus Classification Committee as Severe Acute Respiratory Syndrome Coronavirus 2 (SARS-CoV-2).^[3]^ COVID-19 has become a pandemic over the past year, with 4,962,707 confirmed cases and 326,459 deaths reported in more than 215 countries or areas by May 22, 2020 (data from COVID-19 Dashboard website, Johns Hopkins University).

The epidemiological investigations indicated that the spread of respiratory droplets and close contact with confirmed patients were the main ways of the transmission of SARS-CoV-2. However, some patients with COVID-19 were found with positive SARS-CoV-2 RNA but showed no respiratory symptoms. These asymptomatic infections brought a great challenge to the prevention and control of COVID-19 pandemic. As a result, the accurate and timely confirmation of SARS-CoV-2 infection is extremely important for controlling the spread of the disease. RT-qPCR method for detection of SARS-CoV-2 nucleic acid is the gold standard for the diagnosis of COVID-19.^[4,5]^ SARS-CoV-2 open reading frame 1ab (ORF1ab) and nucleocapsid protein N gene are the main targeted genes for detection. Two consecutive negative results for SARS-CoV-2 RNA, with at least 24 hours’ interval is one of the discharge criteria for patients with COVID-19, according to the guideline of diagnosis and treatment of COVID-19 (Version 5)

RT-qPCR is a rapid method for detecting SARS-CoV-2, but it is inevitable to see the false negative or false positive results which should be screened carefully in clinical laboratory. Some factors may have an influence on nucleic acid detection of SARS-CoV-2, such as RNA extraction method, the sensitivity of the reagent kit, standardization of the sampling process and nucleic acid sequence variation of viral gene loci, and so on. There is an urgent need for the standardization of sampling, transportation, and optimization of RT-qPCR for improving the accuracy of nucleic acid detection.^[1]^ Nevertheless, the nasopharyngeal swab sample for SARS-CoV-2 RNA may become positive for some discharged patients with COVID-19, making it controversial for nucleic acid test of SARS-CoV-2.

Some evidence has clearly showed that asymptomatic infectors of SARS-CoV-2 are also important infectious source.^[6,7]^ In fact, the incubation period of SARS-CoV-2 infection was reported differently, ranging from several hours to more than 14 days.^[8]^ Moreover, patient status, viral load, sampling quality, specimen deliver time, and testing operation process may have influences on the results of SARS-CoV-2 RNA detection. Other factors particularly the age, gender, type of specimen, sampling site, and patient category, may also affect detection results of SARS-CoV-2 RNA.^[9]^ In this study, we analyzed the features of SARS-CoV-2 tests by analyzing results of 12,400 SARS-CoV-2 RNA tests in Wuhan and surrounding areas, the central city of China. We compared the differences of SARS-CoV-2 RNA detection rates in terms of age, gender, specimen type, sampling site, and patient category.

As a result, the retrospective analysis of a large scale SARS-CoV-2 RNA is of great significance for providing clues for revealing the mystery of SARS-CoV-2, which will be helpful for COVID-19 pandemic prevention and treatment, and the etiology and epidemiology of COVID-19 as well.

## 2 Objects and methods

### 2.1 Objects

A total of 12,400 SARS-CoV-2 RNA tests from 6913 individuals were carried out from January 23, 2020 to March 3, 2020 in Renmin Hospital of Wuhan University. The average age of enrolled patients and suspected patients with COVID-19 was 48.86 ± 17.60 years (range from 20 hours to 105 years), with an average age of 51.57 ± 17.57 years (range from 20 hours to 105 years) for 2947 males, 46.85 ± 17.35 years (range from 1day to 98 years) for 3966 females. According to the source of patients, the objects of our study can be divided into 5 groups: general clinic (refers mainly to other common acute and chronic diseases during the pandemic), fever clinic (refers mainly to highly suspicious patients with fever symptoms), community high-risk population (refers to those highly suspicious people who have no suspicious symptoms of COVID-19 but may have a history of close contact or have a history of fever but have not been diagnosed or hospitalized), cabin hospital (a large mobile medical space equipped with medical inspection equipment for basic treatment and monitoring of mild and common COVID-19 patients), and in-patient wards (hospitalized for severe and critically severe COVID-19 patients). The detailed demographic information of the research objects is shown in Table 1. This study was approved by the ethics committee of Renmin Hospital of Wuhan University (WDRY2020-K078) and was exempted from the need for informed consent.

**Table 1:** Comparison of the positive rates of SARS-CoV-2 RNA between genders.

		No. (%) of result			
Gender	No. (%) of cases	Positive	Negative	Presumptive positive	*P* value
Male	2947 (42.63%)	1136 (38.55%)	1662 (56.40%)	149 (5.06%)	<.0001
Female	3966 (57.37%)	1293 (32.60%)	2508 (63.24%)	165 (4.16%)	
Total	6913 (100.00%)	2429 (35.14%)	4170 (60.32%)	314 (4.54%)	

### 2.2 Sample collection and pretreatment

All specimens were collected, transported, and treated as well-protected in accordance with the Expert Consensus for Novel Coronavirus Pneumonia Virus Nucleic Acid Detection, the Novel Coronavirus Testing Laboratory Biosafety Guidelines, and Diagnosis and Treatment Protocol for Novel Coronavirus Pneumonia (Trial version 3 and other related regulations should be implemented. We collected samples from enrolled patients, including upper respiratory tract specimens (oropharyngeal swab, nasopharyngeal swab, nasopharyngeal extracts), lower respiratory tract specimens (sputum, alveolar lavage fluid), and other types of sample including serum, plasma, peripheral venous blood, ocular secretions, urine, stool, breast milk, cerebrospinal fluid and genital secretions. All collected specimens were stored in the clinical laboratory of Renmin Hospital of Wuhan University. SARS-CoV-2 RNA detection was performed within 24 hours after specimen collection. All specimens were incubated at 56°C for 45 minutes in advance for virus inactivation.

### 2.3 Protocol of SARS-CoV-2 RNA detection

The nucleic acid extraction or purification reagent and automated nucleic acid extractor of Ningbo Haiershi Gene Technology Co., Ltd. were used for viral RNA extraction. The 2019-nCoV nucleic acid detection kit (Fluorescence PCR Method, ShuoShi Biotechnology Co., Ltd. and Shengxiang Biotechnology Co., Ltd.) and 7500 Real-Time PCR Systems (Thermo Fisher Scientific) were used for SARS-CoV-2 RNA detection. Both the nucleic acid detection kit from ShuoShi Biotechnology and Shengxiang Biotechnology were double target assay targeting the ORF1ab and N genes, which were the specific conserved sequence encoding the nucleocapsid protein. The results were interpreted as positive only when both the ORF1ab gene and N gene were positive, presumptive positive when any 1 of the 2 genes was positive, and negative when both of these 2 genes were negative. All the presumptive positive patients will be recommended for re-sampling to perform another nucleic acid test. All operations in the clinical laboratory were carried out following the instructions of reagent kits.

### 2.4 Statistical analysis

SPSS 25.0 statistical software was used for data analysis. The quantitative analysis was used to analysis the patients’ general characteristics including gender, age, specimen type, source of patients. The χ^2^ test was used to compare the difference of rates. The measurement data that met the normal distribution were expressed as Mean ± standard deviation (Mean ± SD), and a parametric test was used for comparison among groups. The measurement data that did not meet the normal distribution were expressed as median (interquartile range [IQR]: Q1, Q3), and nonparametric tests were used for comparison among groups. The frequency distribution was used to analyze the window period from the occurrence of clinical symptoms or the close contact to the first positive and the interval time from the first SARS-CoV-2 positive to turning negative after treatment. *P* < .05 was considered as significantly different.

## 3 Results

### 3.1 Comparison of the positive rates of SARS-CoV-2 RNA among subjects of gender and different ages

A total 9927 of 12,400 specimens applying for SARS-CoV-2 RNA testing had complete registration of gender, age, and other necessary information. All of the specimens were collected from 6913 patients, including confirmed patients, suspected patients, and asymptomatic patients with COVID-19. The χ^2^ test showed that positive rate of SARS-CoV-2 RNA detection was significantly higher in male (38.55%, 2947 of 6913) than that of female (32.60%, 3966 of 6913) (χ^2^ = 9.06, 0.01 < *P* < .05). The results were shown in Table 1.

The average age of 6913 patients enrolled in this study was 48.86 years (20 hours–105 years), the average age of male patients (2947) was 51.57 years (20 hours–105 years), female patients (3966) 46.85 years (1day–98 years). There was a significant difference in positive rate of SARS-CoV-2 RNA among different male and female, and various ranges of age (below 5 years, 5–12 years, 12–20 years, 20–40 years, 40–60 years, 60–80 years, and above 80 years), with the highest SARS-CoV-2 positive rate among subjects of 60 to 80 years (51.07%), and the lowest were that of below 5 years (2.13%). The detailed description of SARS-CoV-2 RNA detection results was shown in Table 2.

**Table 2:** Comparison of the positive rates of SARS-CoV-2 RNA among ages.

		No. (%) of results			
Range of ages	No. (%) of cases	Positive	Negative	Presumptive positive	*P* value
≤5	47 (0.68%)	1 (2.13%)	44 (93.62%)	2 (4.26%)	
5–12	17 (0.25%)	2 (11.76%)	13 (76.47%)	2 (11.76%)	
12–20	63 (0.91%)	14 (22.22%)	49 (77.78%)	0 (0.00%)	
20–40	2377 (34.38%)	488 (20.53%)	1803 (75.85%)	86 (3.62%)	<.0001
40–60	2461 (35.60%)	957 (38.89%)	1386 (56.32%)	118 (4.79%)	
60–80	1688 (24.42%)	862 (51.07%)	733 (43.42%)	93 (5.51%)	
≥80	260 (3.76%)	105 (40.38%)	142 (54.62%)	13 (5.00%)	

### 3.2 Comparison of the positive rates of SARS-CoV-2 RNA among different types of specimen

**Figure 1:**
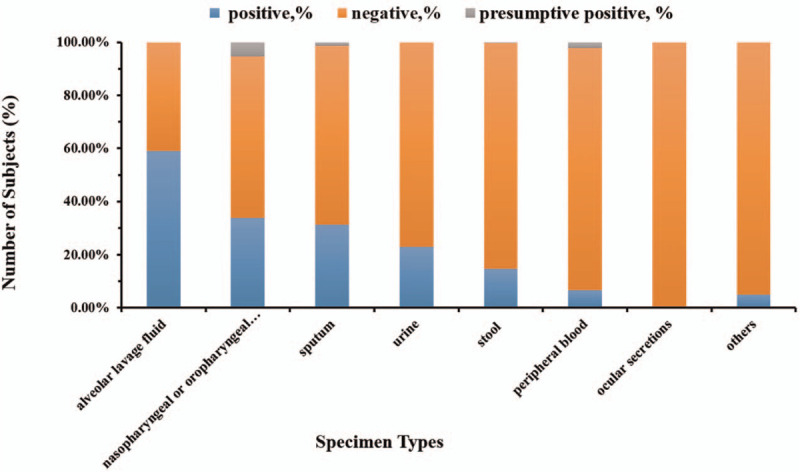
Comparison of the positive rate of SARS-CoV-2 RNA in different types of specimen. We use stacked histogram to depict the composition of the SARS-CoV-2 test results of different types of specimen. The blue bars represent the composition of positive SARS-CoV-2, the pink histograms represent the composition of negative SARS-CoV-2, and the gray bars represent the composition of suspicious positive SARS-CoV-2.

The positive rates of SARS-CoV-2 RNA in different types of specimen were significantly different (χ^2^ = 162.04, *P* < .005) (Fig. 1). While the positive rate of nasopharyngeal or oropharyngeal swab group is significantly different with other group except for the group of urine. The results showed that the alveolar lavage fluid samples had the highest SARS-CoV-2 RNA positive rate (59.09%), followed by nasopharyngeal or oropharyngeal swabs (33.90%), sputum (31.33%), and urine (23.08%). No SARS-CoV-2 RNA was detected in 26 ocular secretions collected in this study. The other groups included 7 serum samples, 3 breast milk samples, 2 cerebrospinal fluid samples, and 8 genital secretion samples. The ORF1ab gene of SARS-CoV-2 virus was detected positive in the breast milk samples. However, SARS-CoV-2 RNA was found negative in serum, cerebrospinal fluid, and genital secretion samples in our study.

### 3.3 Comparison of the positive rates of SARS-CoV-2 RNA among different sources of the subjects

**Figure 2:**
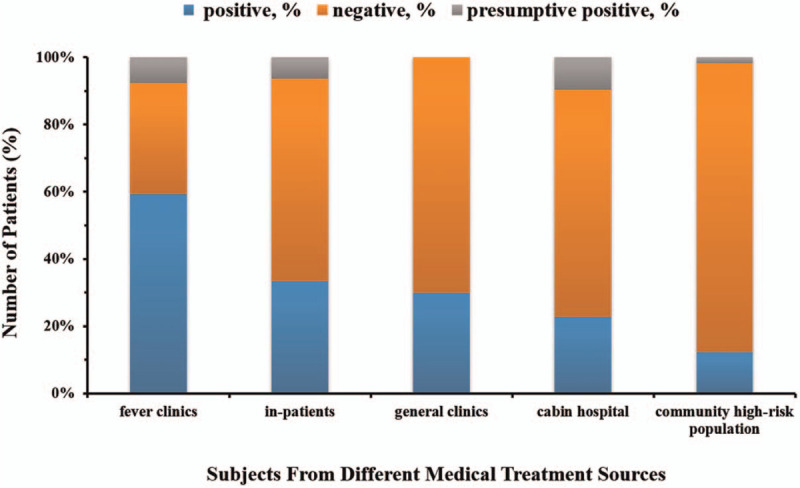
Comparison of SARS-CoV-2 detection results of patients from different sites. We use stacked histogram to depict the composition of the SARS-CoV-2 test results of patients from different source site. The blue bars represent the composition of positive SARS-CoV-2, the pink histograms represent the composition of negative SARS-CoV-2, and the gray bars represent the composition of suspicious positive SARS-CoV-2.

The SARS-CoV-2 RNA positive rates among subjects from different sources were significantly different (*P* < .005). The results showed that the highest positive rate (59.28%) was found in the patients from fever clinic, followed by in-patient (33.42%), cabin hospital (29.91%), community high-risk population (22.73%), and general clinic (12.24%). The results are shown in Figure 2.

### 3.4 Window period of onset SARS-CoV-2 positive detection

**Figure 3:**
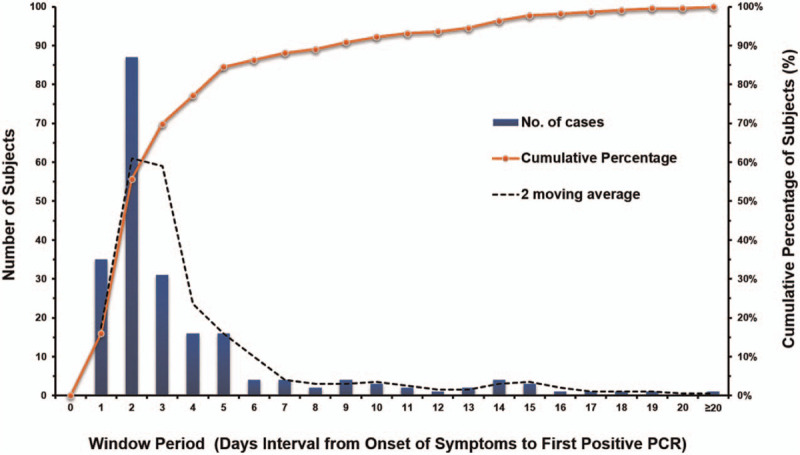
The distribution of window period from the onset of clinical symptoms to the positive result for SARS-CoV-2 RNA. We used a frequency distribution graph to analyze the distribution of the window period from the occurrence of clinical symptoms or the history of close contact to the positive detection of SARS-CoV-2, the X axis represents the window period (days), and the left Y axis represents number of patients with different window period, the right Y axis represents the cumulative percentage of patients number with the increase window period. The blue column represents the number of patients at different window period (days). The solid orange line with dot indicates the cumulative percentage of patients. The dark dotted line represents the 2-period moving average (frequency). It can be seen that the data shows a skewed distribution concentrated on 1 to 5 days window period.

Among the 12,400 tests from 6913 patients, there were many repeated tests of some patients. Among the repeated tests, at least 2 times of consecutive tests was performed, and it was up to 51 times actually. All of the consecutive tests were performed at intervals of 1–2 days. If an originally SARS-CoV-2 positive patient was detected to have 2 consecutive negative nucleic acid tests was defined as turning negative while 2 consecutive positive SARS-CoV-2 RNA tests after becoming negative was defined as relapse. Finally, the time interval from the onset clinical symptoms to the first time of positive SARS-CoV-2 RNA of 219 patients was used to monitor and analysis the window period of onset SARS-CoV-2 positive detection, which could reflect the incubation period of COVID-19. The incubation period refers to the interval between the entry of coronavirus into the host and the appearance of clinical symptoms and signs on the host. The incubation period can be used to formulate the definition of surveillance or case search, infer suspicious exposure time, determine the period of medical observation of close contacts, and even to evaluate whether the spread of the epidemic has ended. Some asymptomatic patients with COVID-19 may remain the status of negative SARS-CoV-2 nucleic acid for a long time. It is the fact that a certain interval exists between the occurrence of clinical symptoms or the discovery of close contact history and the detection of positive SARS-CoV-2 RNA. Our study tracked and analyzed 219 patients’ continuous monitoring results, and found that the median time between the occurrence of clinical symptoms or close contact history and the first appearance of positive SARS-CoV-2 was 2 days (IQR: 1–4 days); 84.47% of patients has 1–5 days of latent time of onset SARS-CoV-2 positive detection, which was a direct evidence indicating the average incubation period (1–5 days) of the COVID-19.^[10]^ The frequency and distribution of the first onset of positive SARS-CoV-2 RNA was demonstrated in Figure 3.

### 3.5 The conversion period of SARS-CoV-2 tests in confirmed patients with COVID-19

572 continuous monitoring COVID-19 patients were tracked and analyzed for the time interval from the first positive to the first turning negative after multiple positive detection, which could indicate the treatment cycle of COVID-19 patients. The time interval of COVID-19 patients’ first turning from negative to turning positive again after repeated negatives is used to represent the patient's relapse cycle.

**Figure 4:**
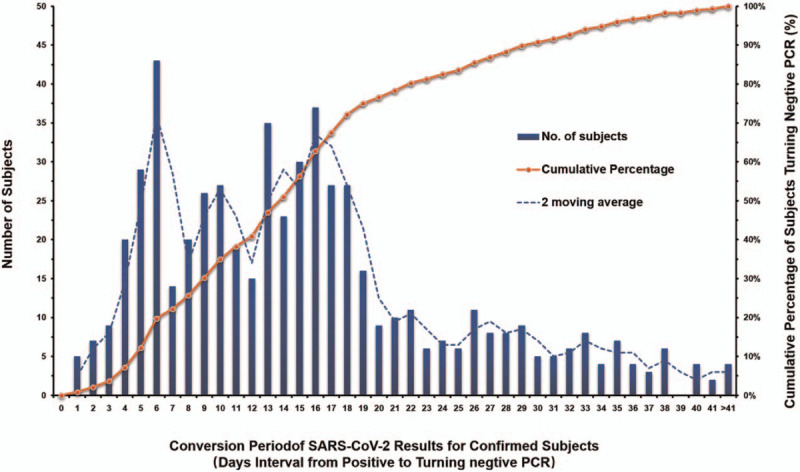
The frequency and distribution of conversion period of SARS-CoV-2 results for confirmed patients. We used a frequency distribution graph to analyze the distribution of the conversion period of SARS-CoV-2 results for confirmed patients, the X axis represents the conversion period of SARS-CoV-2 results for confirmed patients (days), and the left Y axis represents number of patients with different conversion period, the right Y axis represents the cumulative percentage of patients number with the increase conversion period. The blue column represents the number of patients at different conversion period (days). The dotted orange line indicates the cumulative percentage of patients’ number. It can be seen that the data shows an “M-shape” distribution with 2 peaks of conversion period on the 6th and 16th day, respectively.

The median time interval from the first positive to the first turning negative was 14 days (IQR, 8–19.25 days), 72.20% of patients showed the first turning negative in 1–18 days. The frequency distribution chart showed that COVID-19 patients had 2 peaks of turning negative on the 6th and 16th day during treatment, respectively. The SARS-CoV-2 tests of 72 patients (12.59%) turned negative on the 6th day of treatment, and 67 patients (11.71%) turned negative on the 16th day of treatment. The detailed frequency distribution of turning negative time was shown in Figure 4.

## 4 Discussion

This study analyzed the SARS-CoV-2 RNA detection results between male and female patients, and found that the positive rate of SARS-CoV-2 RNA of male patients (38.55%) was significantly higher than that of female patients (32.60%) (*P* < 0.05), which was consistent with the results of other researchers. The difference may be related with higher level of estrogen and immune response, while lower concentrations of angiotensin-converting enzyme 2 in women, which protecting women from the deterioration of SARS-CoV-2 infection. The other reason may be lied with that males are more likely to adopt bad habits of smoking or drink.

Different types of specimens such as nasopharyngeal swab, oral pharyngeal swab, sputum, lower respiratory tract secretions, peripheral blood, and stool can be used for the detection of SARS-CoV-2 RNA in patients with COVID-19, while the detection rate of SARS-CoV-2 RNA among various specimen types has not been studies yet in a large number of samples. Our study analyzed the positive detection rate of 12,400 specimens and found that the detection rate of SARS-CoV-2 in alveolar lavage fluid was the highest (59.09%), followed by nasopharyngeal swabs (33.90%), sputum (31.33%), and urine (23.08%). There is a significantly difference between nasopharyngeal or oropharyngeal swabs group with other group except for urine group.

It is well known that the tropism of SARS-CoV-2 for the respiratory system is sustained by the attachment to angiotensin-converting enzyme 2 highly expressed by lung epithelial cells, which make it reasonable that higher positive rate of SARS-CoV-2 was in lower respiratory tract than that of upper respiratory tract. It seems that the sputum may be the best type of noninvasive specimen when regarding the sampling method, however, most patients with COVID-19 showed symptoms of dry cough, less sputum, while the alveolar lavage only can be collected invasively. We found that nasopharyngeal swab is a simple and feasible way for early clinical screening and diagnosis of COVID-19.

It is worth noting that the positive ORF1ab gene of SARS-CoV-2 was detected in the milk from a pregnant woman, suggesting that SARS-CoV-2 fragments may be transmitted to the fetus through maternal milk, Chinese expert committee suggested that breast feeding should not be recommended for pregnant women who have been diagnosed with COVID-19. In the process of breast feeding, there may be risks such as contact with sexual transmission. It has been confirmed that SARS-CoV-2 may be transmitted from the mother to the fetus through vertical transmission. We found in our study that a variety of specimens could be detected positiveSARS-CoV-2 RNA, which was consistent with clinical findings of multiple damage besides lungs in patients with COVID-19. It has been verified that SARS-CoV-2 may be shed through multiple routes, such as oral-fecal route.^[11,12]^ As a result, physicians should pay more attention to systemic state of patient's whole body during treatment, avoiding cessation of treatment determined solely by the presence of viral nucleic acid in nasopharyngeal swabs.^[13]^

The test of SARS-CoV-2 RNA is the necessary evidence for the diagnosis of COVID-19.^[14]^ We can estimate the incubation period of patient from the time of the first occurrence of respiratory symptoms to the first positive SARS-CoV-2 RNA result. The median time for the incubation period in this study was 2 days (1–4 days), it has been verified that pharyngeal virus shedding was very high during the first week of symptoms, with a peak around day 7 or 10,^[10]^ and the longest incubation period was 40 days, which indicated that physicians should pay great attention to latent asymptomatic infections. It has been confirmed that early testing may increase the probability of diagnosis of COVID-19. Asymptomatic patients in incubation period are latent source of infection of COVID-19. Regardless of the types of specimen, the earlier we collected samples after the occurrence of clinical symptoms, the higher detection rate of SARS-CoV-2 RNA may be found. Detection rates were highest within one week of symptom onset for all tests.

Two consecutive negative SARS-CoV-2 RNA (at least 24 hour's interval) is one of the necessary requirements for discharge of conformed patient with COVID-19 or released patients from isolation. The distribution of time internal for the turning negative of SARS-CoV-2 showed an M-shape picture in our study, with the time interval ranging from 1 day to 51 days. The median conversion period was 14 days. 72.2% of the patients with COVID-19 had a conversion interval of 1–18 days. The M-shape of conversion interval of SARS-COV-2 is possibly due to the relapse of COVID-19 and the false negatives or false positives,^[10]^ and the 2 peaks occurred at around day 7 and day 14, which is coincides with the 14-day's incubation period of COVID-19, which may provide an evidence that the incubation period of the virus affect the treatment cycle of the disease.

A retrospective analysis of the SARS-CoV-2 RNA detection of 12,400 tests with COVID-19 patients and suspected patients showed the actual features of the COVID-19 pandemic. The results of the study showed that men are more susceptible than women, the elder people are more susceptible than the young people. SARS-CoV-2 can be present in multiple organs and tissues of the body and may sustain for a long time. These clinical findings will provide useful support for disease prevention and control of COVID-19. The limitation of this study is that multiple types of specimen of the same confirmed or suspected patients have not been simultaneously detected for SARS-CoV-2 RNA, and we were lack of tracking virus distribution and migration in the infected individual. In addition, this study is a retrospective clinical analysis, and the timeline and treatment measures of patients admitted to hospital were still lacking further analysis. We will further study the relationship between the viral load of SARS-CoV-2, distribution feature and disease progression after SARS-CoV-2 infection.

## Author contributions

**Conceptualization:** Anyu Bao, Jian Gu, Xiaoyun He, Zhijun Zhao, Yongqing Tong.

**Data curation:** Jian Gu, Xiaoyun He, Zhen Chen.

**Formal analysis:** Jingwei Wang, Anyu Bao, Xiaoyun He, Bin Qiao.

**Funding acquisition:** Zhijun Zhao, Jingwei Wang.

**Investigation:** Jingwei Wang, Anyu Bao, Xiaoyun He, Zegang Wu, Bin Qiao, Zhen Chen, Liang Xiong, Yan Zhang.

**Methodology:** Jian Gu, Xiaoyun He, Feng Li, Siqing Mei.

**Project administration:** Zhijun Zhao, Yongqing Tong.

**Resources:** Jingwei Wang, Anyu Bao, Jian Gu, Xiaoyun He, Zegang Wu, Hongyun Zheng.

**Supervision:** Zhijun Zhao, Siqing Mei, Yongqing Tong.

**Validation:** Hongyun Zheng, Siqing Mei, Yongqing Tong.

**Visualization:** Jingwei Wang, Anyu Bao, Jian Gu, Zhijun Zhao.

**Writing – original draft:** Yongqing Tong.

**Writing – review & editing:** Zhijun Zhao, Siqing Mei.
